# Changes in Membrane Lipid Metabolism Accompany Pitting in Blueberry During Refrigeration and Subsequent Storage at Room Temperature

**DOI:** 10.3389/fpls.2019.00829

**Published:** 2019-06-27

**Authors:** Yajuan Wang, Shujuan Ji, Hongyu Dai, Ximan Kong, Jia Hao, Siyao Wang, Xin Zhou, Yingbo Zhao, Baodong Wei, Shunchang Cheng, Qian Zhou

**Affiliations:** College of Food, Shenyang Agricultural University, Shenyang, China

**Keywords:** blueberry, cellular structure, chilling injury, membrane lipidomic, phospholipase D, lipoxygenase

## Abstract

Low-temperature storage is the primary postharvest method employed to maintain fruit quality and commercial value. However, pitting can develop during refrigeration, especially during the shelf life. In this study, a membrane lipidomic approach was employed to analyze the potential relationship between pitting and membrane lipid metabolism during post-cold-storage shelf life. We also determined the changes in ultrastructure and water distribution by low-field nuclear magnetic resonance (LF-NMR) and assessed the permeability of membrane, membrane lipid peroxidation, proline and malondialdehyde contents, and the activity and gene expression of phospholipase D and lipoxygenase, which are involved in membrane lipid metabolism. The results indicated that the changes in blueberry phospholipids during storage could be caused by cold stress. Furthermore, dehydration is a manifestation of chilling injury. Finally, the significant increase in electrolyte leakage, content of malondialdehyde and proline, and activity of phospholipase D and lipoxygenase in chilled blueberry also indicated that membrane lipid metabolism plays an important role in cold stress response.

## Introduction

Blueberry (*Vaccinium* spp.) is popular among consumers and it has high antioxidant activity and health benefits, as it can prevent diseases (Li et al., 2012; Gupta et al., 2015; Chu et al., 2018). The shelf life of blueberry is short at room temperature, but low temperature storage can effectively delay ripening and senescence and inhibit the occurrence of blueberry decay extending its postharvest life. However, pitting developed due to refrigeration, especially during shelf life, greatly affects its commercial value (Zhou et al., 2014). It has been reported that the low temperature above freezing point is one of the three types of temperatures that plants generally experience (Iba, 2002). Tissue damage or death suffered at temperatures above freezing but below 15°C, i.e., chilling injury (CI) (Wolfe, 2010) in fresh agricultural products at low temperature storage has limited the commercial value of several fruits, including Nanguo pear (Shi et al., 2017), loquat (Cao et al., 2011), sweet pepper (Wang et al., 2016), and pineapple (Nukuntornprakit et al., 2015), among others. At the early stage of refrigeration, the physiological characteristics of the cell membrane of fruit and vegetable tissues will change; with the prolongation of refrigeration and transfer to room temperature storage, membrane lipid components will further change and the accumulation of membrane lipid degradation products in fruit and vegetable leads to irreversible membrane lateral phase separation. Eventually, the fruits show a series of CI symptoms (Marangoni et al., 1996).

Lipidomic methods based on liquid chromatography-mass spectrometry (LC-MS) can be used to determine the relative abundance of lipid molecules, thus revealing that changes in membrane lipids play an important role in CI (Tarazona et al., 2015). Some studies have shown that monogalactosyldiacylglycerol (MGDG) and digalactosyldiacylglycerol (DGDG) are the primary components of the chloroplast membrane and the principal contributors to membrane unsaturation as they contain a relatively high level of trienoic fatty acids. It has been reported that a considerable increase in DGDG is a response to low temperature (Gasulla et al., 2016). Additionally, the low temperature response of plants also includes a large increase in phosphatidic acid (PA), lysophosphatidylcholine (LPC), and lysophosphatidylethanolamine (LPE) (Kong et al., 2018). Several studies have indicated that phospholipase D (PLD) is involved in these changes (Bargmann and Munnik, 2006; Hong et al., 2016). To date, there are no studies on the PLD response mechanism to cold stress in blueberry during post-harvest storage. Furthermore, changes in cell membrane also include peroxidation of fatty acids. Several studies have reported that lipoxygenase (LOX) can catalyze lipid peroxidation and increase the unsaturation of plasma membrane lipids, thereby changing membrane fluidity and permeability (Mao et al., 2007).

In the present study, we analyzed the potential relationship between pitting and membrane lipid metabolism during post-cold-storage shelf life. Therefore, we (1) determined the changes in the composition of membrane lipids and ultrastructure, and water distribution by LF-NMR; (2) investigated the permeability of the membrane, and malondialdehyde (MDA) and proline contents; and (3) assessed the activity of PLD and LOX, which are involved in membrane lipid metabolism.

## Materials and Methods

### Fruit Material and Postharvest Treatments

Blueberries (*Vaccinium* spp. “DuKe”) were harvested from a commercial orchard located in Shenyang, Liaoning Province, China, and transported to the laboratory in the Shenyang Agriculture University within 2 h. Blueberries of uniform size, color, maturity (80∼90%), and presenting no mechanical injury were selected. The fruits were precooled at 0 ± 0.5°C for 10 h. Subsequently, one fifth of the fruits were stored at 20 ± 0.5°C under 80% relative humidity (RH) for up to 8 days as the control group. The remaining blueberries were stored at a low temperature (0 ± 0.5°C) for 15, 30, 45, and 60 days and stored at 20 ± 0.5°C under 80% RH for 8 days. Three biological replicates were assessed. Three biological replicates were performed on three batches of blueberries harvested at different times and they were treated at the same way. Each biological replicate was composed of 20 Kg blueberry fruits and then divided into small boxes, each containing 125 g, totaling 160 boxes.

### Measurement of Fruit Pitting Incidence

Pitting incidence was measured after 0, 15, 30, 45, and 60 days cold storage at 0 ± 0.5°C following by 8 days of shelf life at 20 ± 0.5°C with 80% RH. Pitting incidence was determined as:

Pittingincidence=N/oN×100%,

where N_o_ is the number of pitted blueberries and N is the total number of blueberries.

### Membrane Lipid Analysis

Membrane lipids were extracted as described in Welti et al. (2002) with some modifications. Blueberry tissues were soaked in dimethylcarbinol, containing 0.01% butylated hydroxytoluene (BHT), preheated at 75°C and then in water bath for 15 min at 75°C. Chloroform (1.5 mL) and ultra-pure water (0.6 mL) were then successively added to the samples. The mixture was evenly blended and kept away from light at room temperature. After shaking for 1 h at 150 g min^–1^, the extract was transferred into a new glass tube. Subsequently, 4 mL CHCl_3_/MeOH (2:1, v/v) (0.01% BHT) were added to the samples and these were shaken for 30 min at 150 g min^–1^. The extract was transferred into new glass tubes, and the process above was repeated five times, including the isopropanol extraction. All the extracts were mixed, 1 mL 1 M KCL was added, samples were centrifuged at 500 *g* min^–1^ for 5 min, and the water phase was discarded. After adding 2 mL of ultra-pure water to the extract, this was centrifuged at 500 *g* min^–1^ for 5 min, and the aqueous phase was discarded. Finally, the extract was dried with nitrogen gas (N_2_) and stored at –80°C.

Membrane lipids were measured as described in Xiao et al. (2010).

### Fatty Acid Analysis

Fatty acids were measured as described in Zhang and Tian (2010).

Gas chromatography column was HP 19091F-102 (25 m × 200 μm × 0.30 μm nominal) fused silica capillary column and gasification chamber temperature was 250°C. Programmed heating, initial oven temperature of 80°C, which was increased by 10°C min^–1^ until 200°C and then increased by 5°C min^–1^ to 230°C and maintained for 5 min. The carrier gas was helium, the injection volume was 1 μL, and the split ratio was 20:1. Additionally, the solvent delay was 3 min. Mass spectrometry was electron impact ion source (EI). The ion source temperature was 230°C, electron energy was 70 eV, multiplier voltage was 1235 V, and the scan range, m/z was 12–550.

Identification of fatty acids was based on NIST/Wiley MS Search 2.0 mass spectral libraries. The fatty acids were quantified using the external standards and than expressed in relative concentration.

### Ultrastructure Observations

The mesocarp of blueberry (1 mm × 1 mm × 3 mm) was rapidly fixed in 2.5% glutaraldehyde (v/v) for 12 h at 4°C. The sample was fixed in 1% osmium tetroxide for 2 h, and then washed three times with 0.1 M phosphate buffer (pH 7.2). A series of increased ethanol and acetone concentrations were then used to dehydrate the samples (each concentration was applied for 15 min). Additionally, a 100% acetone solution was used to dehydrate samples for 30 min. The sample was immersed in a mixture of SPI-812 embedding medium and propylene oxide, and then immersed in the embedding medium only for 12 h. All samples were observed using Hitachi HT7700 transmission electron microscope (Hitachi, Japan).

### LF-NMR Measurements

The 23.3 MHz NMR Analyzer MesoMR23-060H-I (Niumag Co., Ltd., China) was used for water distribution analysis. Transverse relaxation time (T2) and magnetic resonance imaging detection of samples were measured by the Carr-Purcell-Meiboom-Gill (CPMG) sequence and the spin echo sequence, respectively. For NMR imaging, the redder the color of the sample images, the more water they contained.

### Measurement of Electrolyte Leakage and MDA and Proline Contents

Electrolyte leakage was measured according to Zhu et al. (2009), and expressed as relative conductivity (%) to reflect membrane permeability.

Blueberry tissue (1 g) was added to 5 mL of 10% (w/v) trichloroacetic acid (TCA). After grinding the homogenate, the blueberry tissue was centrifuged at 4°C and 10,000 × *g* for 20 min. After collecting the supernatant (2 mL), 2 ml of 0.67% (w/v) thiobarbituric acid (TBA) were added, mixed, and boiled in water for 20 min. After this period, samples were removed from the water bath and centrifuged again after cooling. The absorbance values of the supernatant at 450, 532, and 600 nm were determined. The experiment was repeated three times. The MDA content was determined as:

$$\text{MDA}\,\left({\text{nmol g}}^{-1}\,\text{FW}\right)=\left[6.45\times \left(A_{532}-A_{600}\right)-0.56\times A_{450}\right]\times 5$$

The proline content was measured as described in Zhao et al. (2009) and expressed as μg kg^–1^ fresh weight (FW).

### Determination of PLD and LOX Activities

Blueberry tissue (5 g) was homogenized in 5 mL of pre-cooled phosphate-buffered saline (PBS) (0.1 M Na_2_HPO_4_⋅12H_2_O, 0.1 M NaH_2_PO_4_⋅2H_2_O) on ice and centrifuged for 20 min at 4°C and 8300 × *g*. The supernatant was collected and the activitiy of PLD was determined according to the instructions provided in the Plant Phospholipase D ELISA Kit.

Blueberry tissue (5 g) was homogenized in 5 mL of pre-cooled acetate buffer [4% (m/v) polyvinyl-poly-pyrrolidone, 1% (v/v) Triton X-100] on ice and centrifuged at 12,000 × *g* for 20 min at 4°C. The supernatant was collected and the activitiy of LOX was determined according to the instructions provided in the Plant Lipoxygenase ELISA Kit.

### Quantitative Real-Time PCR of *PLD* and *LOX*

The extraction of total RNA from blueberry tissues and cDNA synthesis were performed according to the manufacturer’s instructions of the OminiPlant RNA Kit (CWBIO, China) and HiFiScript cDNA Synthesis Kit (CWBIO, China), respectively. The primers used in the quantitative real-time PCR (qRT-PCR) (Table 1) were designed in Primer Premier 5.0. The qRT-PCR was performed according to the manufacturer’s instructions of the UItraSYBR Mixture (Low Rox) Kit (CWBIO, China).

**TABLE 1:** Primers used in the quantitative real-time PCR assays.

**Gene**	**Forward**	**Reverse**
*Actin*	5′-ACTACCATCCACTCTATCACCG-3′	5′-AACACCTTACCAACAGCCTTG-3′
*PLDα1*	5′-CGAGACATTCAGCCATCCAA-3′	5′-AAGCAGGTGACCAGGCAGAT-3
*PLDα2*	5′-TCTGGCGGAAAGTATTATGAGC-3′	5′-CTGTAACCACCGACCACCCTGT-3′
*PLDβ*	5′-TCAGCTTACGTCGTTATTCCTATGTG-3′	5′-ACGGTTGCCAAGACAGTAGAAGTTC-3′
*PLDδ*	5′-ATGATACTCCTGAGCATCGGTTGTTC-3′	5′-ATGGTTGCCTTGGAGCCTTACTTC-3′
*LOX1*	5′-GGATCACCATGATGCGCTAA-3′	5′-ATGGCTTCAGTGTTCCGTCA-3′

### Statistical Analyses

Statistical analyses and data plotting were performed using SPSS version 20.0 software (SPSS Inc., United States) and Origin 8.0, respectively. One-way analysis of variance (ANOVA) and Duncan’s multiple comparison tests were used to analyze the data. But pitting incidence of blueberry fruits was analyzed by non-parametric statistics (Kruskal–Wallis test). Differences were considered significant at *P* < 0.05.

## Results

### Pitting Incidence of Blueberry Fruits

**FIGURE 1:**
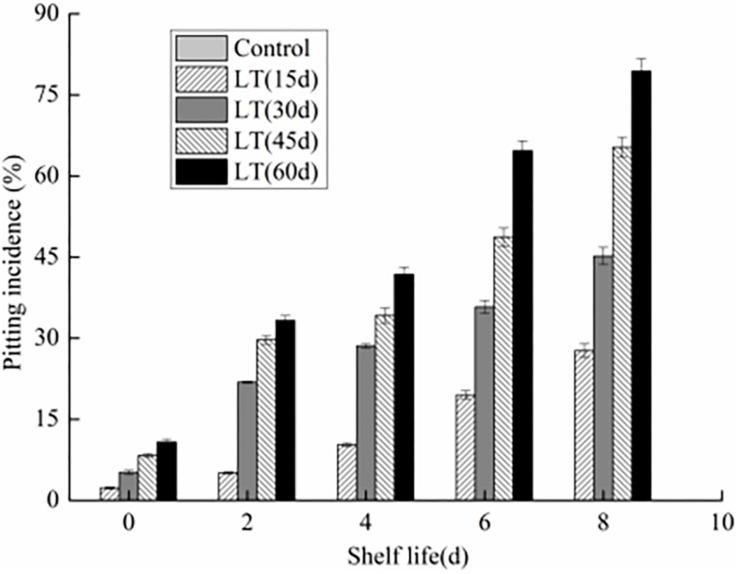
Changes in pitting incidence in blueberries during shelf life at room temperature. Control is the fruits stored at room temperature; LT (15 days), LT (30 days), LT (45 days), and LT (60 days) are fruits held at 0 ± 0.5°C for 15, 30, 45, and 60 days, respectively, prior to storage at room temperature. Mean ± SE of three replicate experiments are shown.

We observed that there are no pitting symptoms in blueberries stored directly at 20 ± 0.5°C with 80% RH for 8 days (Figure 1). However, chilling injury symptoms, characterized by pitting was observed in blueberry fruits cold storage after 15 days. Meanwhile, with the extend of the cold storage, the pitting incidence was also increasing (*P* < 0.05). Furthermore, the pitting of blueberry fruits was aggravated with the extension of shelf life after refrigeration (*P* < 0.05). After 45 and 60 days refrigeration followed by an 8 days exposure to 20 ± 0.5°C with 80% RH, the pitting incidence increased significantly to 65.3 and 79.4%, respectively (*P* < 0.05).

### Modification of Membrane Lipid Composition

**FIGURE 2:**
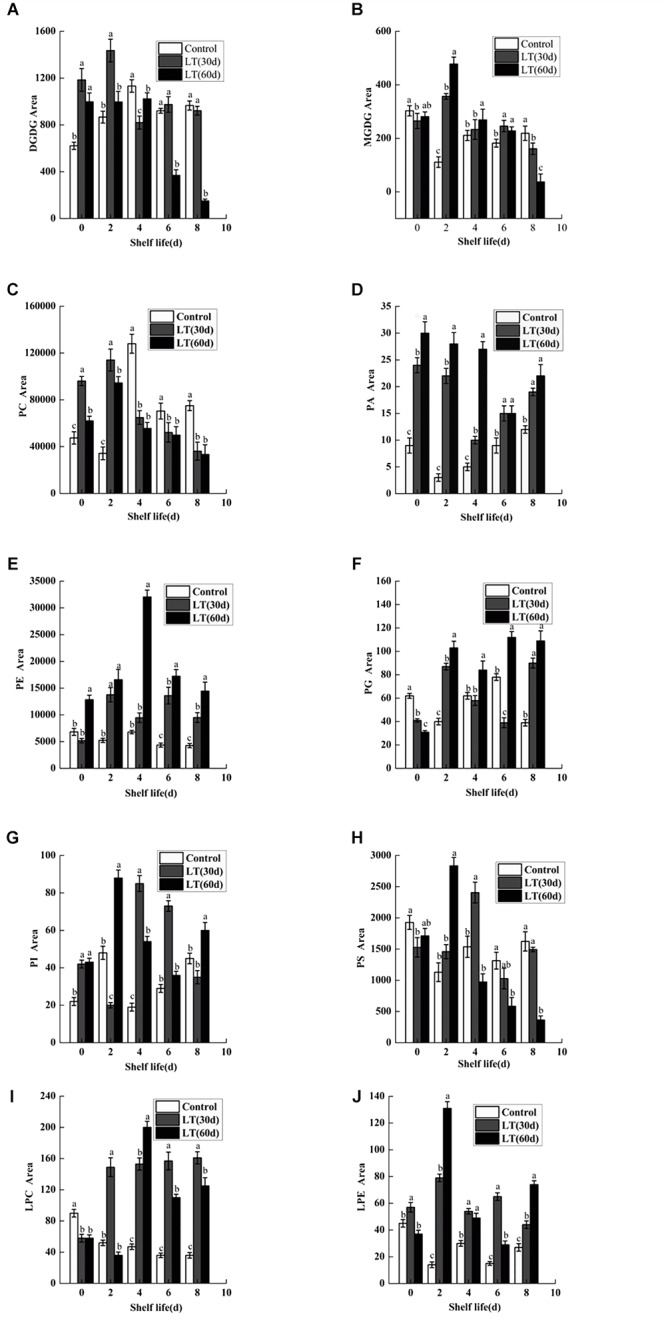
Changes in the content of total DGDG **(A)**, MGDG **(B)**, PC **(C)**, PA **(D)**, PE **(E)**, PG **(F)**, PI **(G)**, PS **(H)**, LPC **(I)**, and LPE **(J)** in blueberries during shelf life at room temperature. Control is the fruit stored at room temperature; LT (30 days) and LT (60 days) are fruits maintained at 0 ± 0.5°C for 30 and 60 days, respectively, prior to storage at room temperature. Mean ± SE of three replicate experiments are shown. The letters a and b show significant differences according to the independent sample *t*-test (*P* < 0.05) at each time point.

Ten kinds of lipids, including MGDG, DGDG, PA, phosphatidylcholine (PC), phosphatidylethanolamine (PE), phosphatidylglycerol (PG), phosphatidylinositol (PI), phosphatidylserine (PS), LPC, and LPE were obtained from blueberry tissues by the lipidomic approach using an automated electrospray ionization-tandem mass spectrometer. As shown in Figure 2A, and compared with that on the harvest day, a significant increase in DGDG was observed in fruits refrigerated for 30 and 60 days. Furthermore, a significantly (*P* < 0.05) higher level of DGDG was observed in fruits cold stored for 30 days than in those stored for 0 or 60 days on day 2 of shelf life. When cold stored for 60 days, the MGDG level in blueberry increased significantly, and on day 2 of shelf life, it reached the maximum level. The MGDG level then declined continuously thereafter and it was significantly lower than that in fruits cold stored for 0 and 30 days on day 8 of shelf life (Figure 2B). On the last days of shelf life, the PC content of blueberries that were cold stored was significantly lower than that of non-refrigerated blueberries (Figure 2C). The PA content of blueberries that were cold stored was higher than that of non-refrigerated blueberries during shelf life. The increase of PA during shelf life appeared to be related to the duration of refrigeration. On the first 4 days of shelf life, the PA content was significantly (*P* < 0.05) higher in blueberries cold stored for 60 days compared with that of blueberries cold stored for 0 and 30 days (Figure 2D). There were no significant differences in the PE content of blueberries held in short-term cold storage for up to 30 days (Figure 2E). In contrast, the PE content of blueberries cold stored for 60 days increased significantly, and on day 4 of shelf life, it reached the maximum level and declined continuously thereafter. A significant (*P* < 0.05) decrease in PG content was observed during cold storage; however, the PG content of blueberries maintained in cold storage for 60 days increased after 2 days of shelf life to significantly higher levels than that observed in fruits stored for 0 and 30 days (Figure 2F). After refrigeration, the PI content of blueberries increased significantly, and the PS content decreased slightly. However, both of them reached a significant high level in blueberries stored for 30 days on day 2 of shelf life (Figures 2G,H). Compared with the level on the harvest day, a slight decrease in LPE was observed in blueberries cold stored for 60 days. However, this level was significantly higher than that observed in blueberries stored for 0 and 30 days on day 2 of shelf life (Figure 2J). We also observed that the LPC content was significantly (*P* < 0.05) higher in blueberries refrigerated for 60 days than in those stored for 0 and 30 days on day 4 of shelf life (Figure 2I).

### Fatty Acid Analysis

**FIGURE 3:**
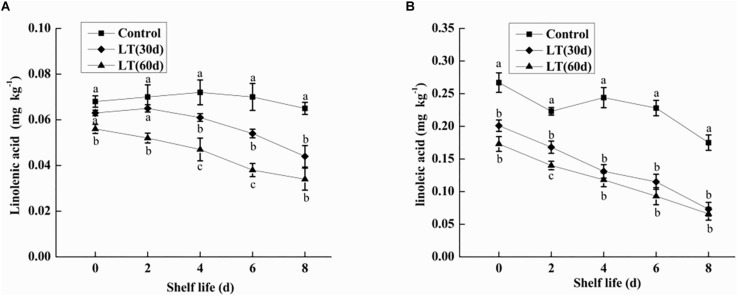
Changes in linolenic acid **(A)** and linoleic acid **(B)** content in fruits during shelf life at room temperature. Control is the fruit stored at room temperature; LT (30 days) and LT (60 days) are fruits maintained at 0 ± 0.5°C for 30 and 60 days, respectively, prior to storage at room temperature. Mean ± SE of three replicate experiments are shown. The letters a and b show significant differences according to the independent sample *t*-test (*P* < 0.05) at each time point.

Linolenic acid is a polyene fatty acid containing three conjugated double bonds and it is a substrate of LOX. As shown in Figure 3A, no significant changes were observed in linolenic acid content after 30 days of cold storage compared with the level on the day of harvest. However, the content of linolenic acid in blueberries decreased significantly (*P* < 0.05) after 60 days of cold storage, and the content of linolenic acid was as low as 0.056 mg kg^–1^. Additionally, during the whole shelf life, the content of linolenic acid showed a significantly downward trend, and maintained a significantly (*P* < 0.05) lower level than in other periods.

Linoleic acid is a fatty acid containing two unsaturated double bonds and it is also a substrate of LOX. As shown in Figure 3B, the content of linoleic acid in blueberries decreased with the increase of cold storage time, and it was significantly lower than that on the day of harvest (*P* < 0.05). Compared with non-refrigerated blueberries, the linoleic acid content of refrigerated blueberries was significantly (*P* < 0.05) decreased during shelf life at room temperature, and the decrease of linoleic acid increased with the prolongation of refrigeration time.

### Ultrastructural Changes in Blueberries

**FIGURE 4:**
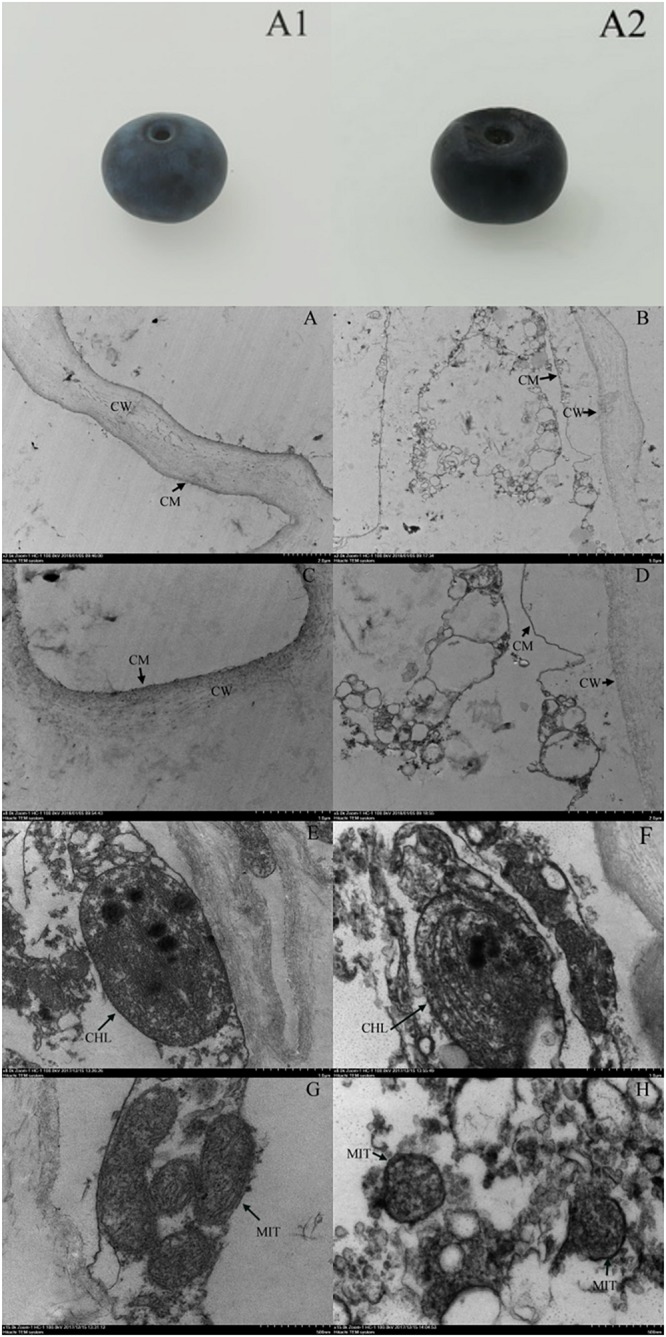
Apparent changes in fresh blueberry on the day of harvest **(A1)**, blueberry on the fourth day of shelf life after 30 days of cold storage (with pitting) **(A2)**. Ultrastructural changes in fresh blueberry on the day of harvest **(A,C,E,G)** and blueberry on the fourth day of shelf life after 30 days of cold storage (with pitting) **(B,D,F,H)**. CW, cell wall; CM, cell membrane; CHL, chloroplast; MIT, mitochondria.

We observed no apparent symptoms of pitting in fresh blueberries on the day of harvest (Figure 4A1). However, pitting developed due to refrigeration, especially during shelf life (Figure 4A2). The cell structure of the fresh blueberries was intact, and the cell membrane was close to the cell wall (Figures 4A,C). However, the cell wall of the pitted blueberries on day 4 of shelf life after 30 days of cold storage was narrowed, and the cell membrane was wrinkled and it induced cells plasmolysis. We also observed the increase of vesicles and the leakage of contents in pitted blueberries (Figures 4B,D). In the fresh blueberries, the organelles were well structured, with many layered thylakoids and a few osmiophilic granules in chloroplasts (Figure 4E), and mitochondria were abundant and normal in morphology with the bilayer membrane structurally intact (Figure 4G). However, after refrigeration, the chloroplast cell wall of blueberry was damaged, the thylakoids were enlarged, the structure of grana lamellae was scattered, and a large number of black flocculent substances appeared around them (Figure 4F). In addition, the number of mitochondria in blueberries with pitting decreased, and the mitochondria were swelled, with blurred internal cristae structure, and some mitochondrial membranes began to rupture and gradually disintegrated (Figure 4H).

### Water Distribution and NMR Image Analysis

**FIGURE 5:**
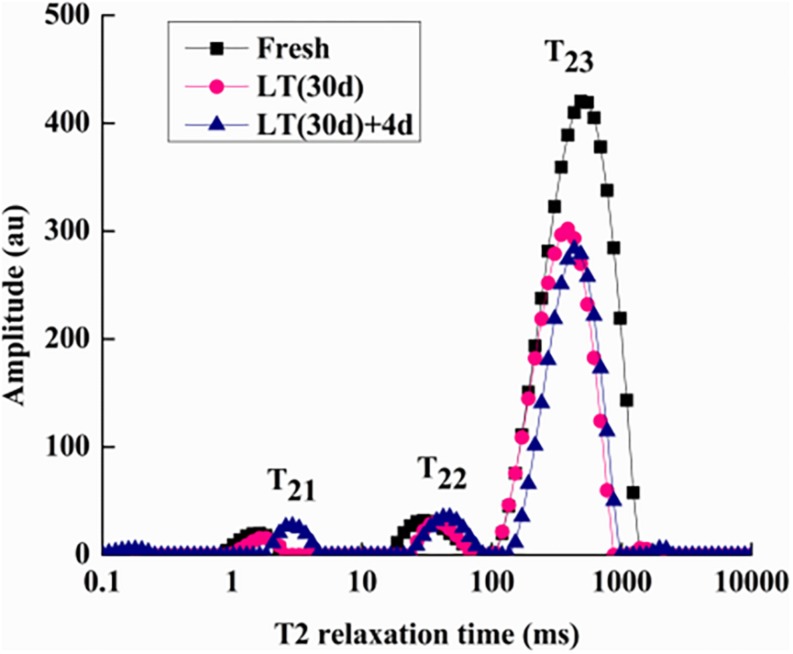
Low-field nuclear magnetic resonance T2 relaxation time curves of blueberries during storage. Fresh is the fruit sample obtained on the day of harvest, LT (30 days) is the fruits cold stored for 30 days, and LT (30 days) + 4 days is the fruits on the fourth day of shelf life after 30 days of cold storage (with pitting). T21 and T22 represent the transverse relaxation times of the bound water molecules which are tightly combined with organic matter like proteins. T23 refers to the transverse relaxation time of free water molecules. The experiment was repeated three times. To clearly indicate the distribution of water in blueberries, a representative set of data was shown in the figure.

To determine internal water changes, we monitored T_2i_ relaxation time and the corresponding water population M_2i_ (Table 2) in blueberries. As shown in Figure 5, all the samples had three proton fractions: T_21_, T_22_, and T_23_. The fastest relaxation component, T_21_ (0.1 < T_21_ < 10 ms), was considered as bound water. For fresh samples, the proton population (M_21_) was 2.23%, and it increased to over 4.75% in LT (30 days) + 4 days samples. The second relaxation component, T_22_ (10 < T_21_ < 100 ms) was regarded as immobilized water, and, compared with fresh blueberries, the M_22_ of LT (30 days) samples and LT (30 days) + 4 days samples increased significantly (*P* < 0.05). The third relaxation component, T_23_ (100 < T_21_ < 1000 ms) was identified as free water and its proportion (M_23_) was 93.16, 91.55, and 87.13% in fresh, LT (30 days), and LT (30 days) + 4 days samples, respectively.

**TABLE 2:** Changes in the relative amount of water (M2i) at the corresponding relaxation times (T2i) in blueberries during storage.

**Treatment condition**	***M*_21_ (%)**	***M*_22_ (%)**	***M*_23_ (%)**
Fresh	2.23 ± 0.05^b^	4.61 ± 0.02^c^	93.16 ± 0.06^a^
LT (30 days)	2.66 ± 0.30^b^	5.60 ± 0.33^b^	91.55 ± 0.36^a^
LT (30 days) + 4 days	4.75 ± 0.19^a^	7.71 ± 0.06^a^	87.13 ± 1.14^b^

*Fresh corresponds to blueberries obtained on the day of harvest, LT (30 days) corresponds to blueberries cold stored for 30 days, LT (30 days) + 4 days corresponds to blueberries on the fourth day of shelf life after 30 days of cold storage (with pitting). Values are mean ± SD. Values in the same column with different letters are significantly different (*P* < 0.05).*

**FIGURE 6:**
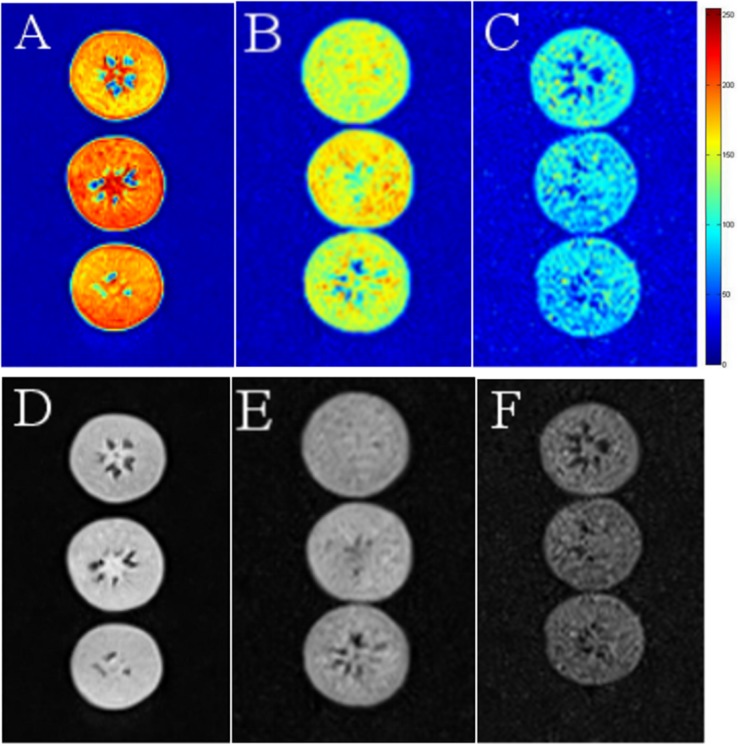
Nuclear magnetic resonance images of fresh **(A,D)**, LT (30 days) **(B,E)**, and LT (30 days) + 4 days **(C,F)** samples. The images were color coded based on their grayscale value. Red corresponds to the “bright part” and blue to the “dark part.” Fresh is the fruit sample on the day of harvest, LT (30 days) is the fruits cold stored for 30 days, and LT (30 days) + 4 days is the fruits on the fourth day of shelf life after 30 days of cold storage (with pitting). The experiment was repeated three times. To clearly indicate the moisture content in blueberries, a representative set of data was shown in the figure.

We used MRI to directly detect water distribution and proton mobility in tissues. The fresh blueberries were mostly red in NMR images, indicating their high moisture content (Figures 6A,D). However, in the LT (30 days) and LT (30 days) + 4 days samples, the red area decreased, and the images of LT (30 days) + 4 days samples mostly presented blue areas, which indicated reduced moisture content (Figures 6B,C,E,F).

### Electrolyte Leakage and MDA and Proline Contents

**FIGURE 7:**
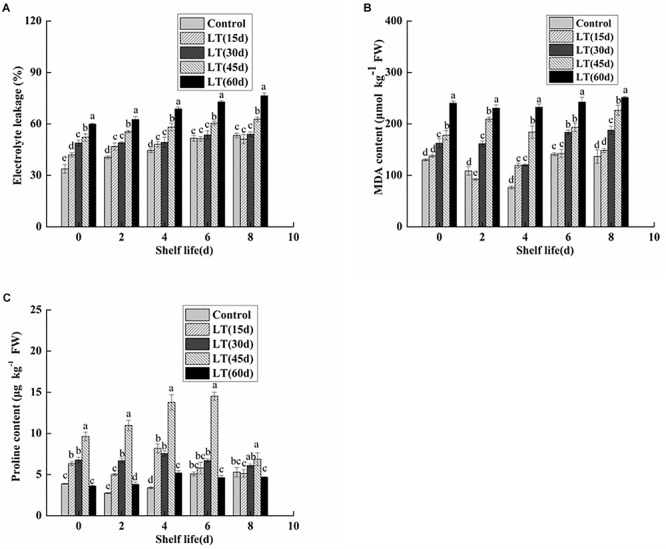
Physiological changes in electrolyte leakage **(A)**, MDA content **(B)**, and proline content **(C)** in blueberries during shelf life at room temperature. Control is the fruit stored at room temperature; LT (15 days), LT (30 days), LT (45 days), and LT (60 days) are fruits held at 0 ± 0.5°C for 15, 30, 45, and 60 days, respectively, prior to storage at room temperature. Mean ± SE of three replicate experiments are shown. The letters a and b show significant differences according to the independent sample *t*-test (*P* < 0.05) at each time point.

Electrolyte leakage is an index reflecting the changes of cell membrane permeability and the degree of damage to cell membrane. As shown in Figure 7A, electrolyte leakage in blueberries showed an increasing trend with the extension of cold storage time (*P* < 0.05). Furthermore, the electrolyte leakage of blueberries cold stored for 45 and 60 days was significantly (*P* < 0.05) higher throughout shelf life. It was shown that the permeability of blueberry cell membranes changed significantly under long-term low temperature environment, and that the integrity and stability of cell membranes were damaged. Furthermore, the damage of cell membranes was aggravated with the extension of shelf life after refrigeration.

As shown in Figure 7B, the MDA content was low in non-refrigerated blueberries throughout the shelf life. In contrast, the MDA content showed an increasing trend and almost doubled during 60 days of cold storage. Moreover, the MDA content of cold-stored blueberries was higher than that of non-refrigerated blueberries during shelf life. A significant (*P* < 0.05) increase was also observed in blueberries cold stored for 60 days compared with that of blueberries cold stored for 30 and 45 days. These data clearly indicated that the increase in the MDA content during shelf life might be related to the duration of cold storage.

Free proline content in fruit and vegetable tissues increases significantly under low temperature stress. As shown in Figure 7C, the proline content remained low in blueberries that were not refrigerated. We also observed that the proline content of cold-stored blueberries increased and then decreased during both cold storage and shelf life. Additionally, for blueberries after 45 days of refrigeration, the proline content was significantly higher than that of other blueberries during the 6-day shelf life (*P* < 0.05).

### PLD and LOX Activities

**FIGURE 8:**
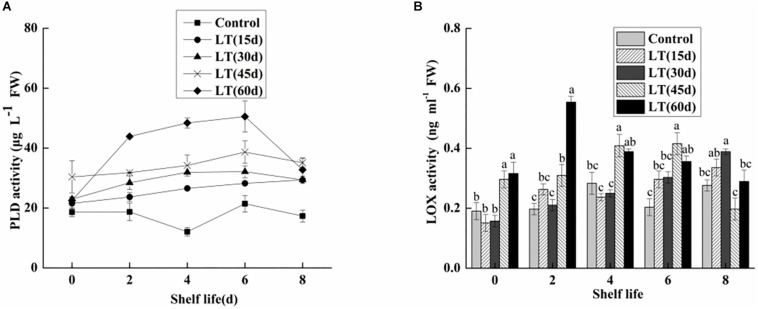
Physiological changes in PLD activity **(A)** and LOX activity **(B)** in blueberries during shelf life at room temperature. Control is the fruits stored at room temperature; LT (15 days), LT (30 days), LT (45 days), and LT (60 days) are fruits held at 0 ± 0.5°C for 15, 30, 45, and 60 days, respectively, prior to storage at room temperature. Mean ± SE of three replicate experiments are shown. The letters a and b show significant differences according to the independent sample *t*-test (*P* < 0.05) at each time point.

Phospholipase D is commonly expressed in fruits and vegetables. It is the starting enzyme of the lipid catabolic pathway and therefore a key element of this pathway (Sun et al., 2011). Additionally, PLD also plays a significant role in transmembrane signal transduction and cell regulation, including response to adverse stress and aging processes in postharvest fruit and vegetables. As shown in Figure 8A, changes in the PLD activity of blueberries during different cold storage times were observed. The PLD activity was low in non-refrigerated blueberries during all shelf life. When the fruits were maintained at 0°C, the PLD activity initially increased, and then decreased. The maximum value was 9.054 μg L^–1^ FW in fruits refrigerated for 45 days, and this was significantly (*P* < 0.05) higher than in other blueberries. With the exception of day 8 of shelf life, the PLD activity was maintained at a significantly (*P* < 0.05) higher level in fruits transported from cold storage for 60 days to room temperature.

As shown in Figure 8B, there were no obvious changes in the LOX activity of blueberries refrigerated for up to 30 days. In contrast, the LOX activity of blueberries stored for 45 and 60 days increased by 0.2963 and 0.31614 μg L^–1^ FW, respectively. The results showed that low temperature storage could inhibit the increase of LOX activity in short-term cold-stored blueberry fruits. However, the LOX activity began to increase in blueberry fruits transferred to room temperature after cold storage for 30 days, which was closely related to the occurrence of pitting in blueberries. Additionally, in fruits cold stored for 60 days, the LOX activity increased sharply and peaked on day 2 of shelf life, and then decreased. These results clearly show that LOX activity was significantly increased in the late stage of cold storage, and that the membrane lipid oxidation of blueberries is intensified, which leads to the intensification of pitting.

### *PLD* and *LOX* Gene Expression Analysis

**FIGURE 9:**
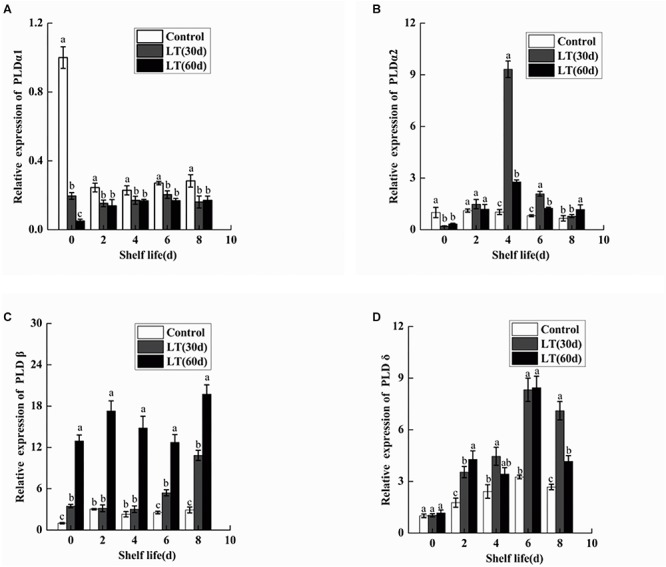
Changes in the gene expression of *PLDα1* **(A)**, *PLDα2* **(B)**, *PLDβ* **(C)**, and *PLDδ* **(D)** in blueberries during shelf life at room temperature. Control is the fruits stored at room temperature; LT (30 days) and LT (60 days) are fruits held at 0 ± 0.5°C for 30 and 60 days, respectively, prior to storage at room temperature. Mean ± SE of three replicate experiments are shown. The letters a and b show significant differences according to the independent sample *t*-test (*P* < 0.05) at each time point.

To determine the gene expression of *PLDα1*, *PLDα2*, *PLDβ*, and *PLDδ*, qRT-PCR was performed. As shown in Figure 9A, the expression of *PLDα1* in blueberry fruit decreased significantly (*P* < 0.05) during cold storage. Furthermore, the expression of *PLDα1* showed lower level during the shelf life of refrigerated than non-refrigerated blueberries (*P* < 0.05). Similarly, the expression of *PLDα2* decreased in blueberries stored at 0°C (*P* < 0.05). However, in fruits cold stored for 30 days, the expression of *PLDα2* increased sharply on day 4 of shelf life, and it was significantly (*P* < 0.05) higher than that in fruits cold stored for 60 days and in non-refrigerated fruits (Figure 9B). Conversely, the expression of *PLDβ* increased in blueberries stored at 0°C (*P* < 0.05). Additionally, in fruits cold stored for 60 days, the expression of *PLDβ* increased sharply and remained at a significantly (*P* < 0.05) higher level during all shelf life (Figure 9C). Compared with the level on the day before cold storage, there was no significant change in the expression of *PLDδ* during cold storage. However, in fruits cold stored for 30 and 60 days, the expression of *PLDδ* exhibited a similar tendency, with peak expression on day 6 (*P* < 0.05), and than decreased (Figure 9D).

**FIGURE 10:**
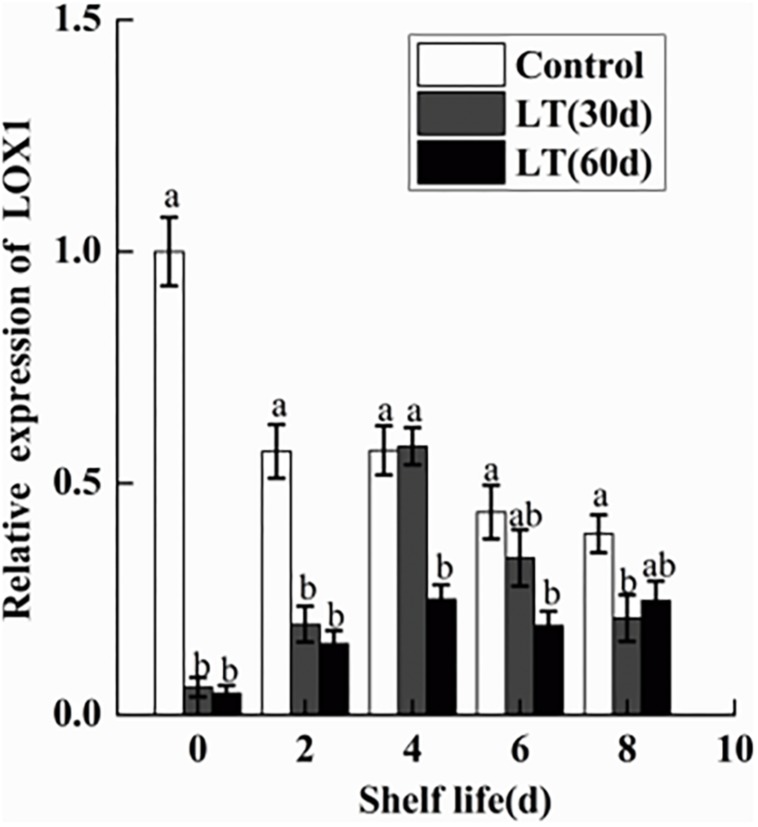
Changes in the gene expression of *LOX1* in blueberries during shelf life at room temperature. Control is the fruits stored at room temperature; LT (30 days) and LT (60 days) are fruits held at 0 ± 0.5°C for 30 and 60 days, respectively, prior to storage at room temperature. Mean ± SE of three replicate experiments are shown. The letters a and b show significant differences according to the independent sample *t*-test (*P* < 0.05) at each time point.

As shown in Figure 10, a significant (*P* < 0.05) decrease in the expression of *LOX1* was observed in blueberries during cold storage. However, in fruits cold stored for 30 and 60 days, *LOX1* was activated and its expression exhibited a similar tendency, with peak expression on day 4 of shelf life (*P* < 0.05), followed by a decrease until day 8. Therefore, the expression of *LOX1* appeared to be closely related to the pitting of blueberries.

## Discussion

Low-temperature storage is the primary postharvest method employed to maintain fruit quality and its commercial value (Laura et al., 2010). In the present study, blueberries were stored at low temperature (0 ± 0.5°C) for 15, 30, 45, and 60 days. This method can preserve the quality and extend the postharvest life of fruits. However, pitting developed due to refrigeration, especially during shelf life; the longer the blueberries are refrigerated, the more serious the pitting is. It has been reported that the phospholipid content of cell membranes will change with the severity of the low temperature stress experienced by plants in order to adapt to changes in the external environment (Welti et al., 2002). In the present study, we observed a higher level of DGDG after refrigeration, which is consistent with previous findings that conical MGDG molecules can be easily converted to cylindrical DGDG molecules to maintain the stability of thylakoid membranes when exposed to low temperature and osmotic stresses (Kong et al., 2018). Additionally, the accumulation of PI also provided evidence of membrane lipid remodeling due to cold stress. Some researches have shown that the accumulation of PI under desiccation can enhance the stability of membrane structure (Gasulla et al., 2016). We also observed the lower level of PC and higher levels of PA, PE, PS, and PG after cold storage. Some studies have shown that low temperature stress can activate the degradation of phospholipids by PLD, resulting in a large number of PA molecules. In addition, PLD can also catalyze the synthesis of PE, PS, and PG using the large amount of PC through phosphoryl transfer (Bargmann and Munnik, 2006). Previous studies have also found that LPC and LPE accumulate significantly under low temperature stress, therefore playing a significant role in signaling when the plant is under cold stress (Tarazona et al., 2015). It is worth mentioning that the increase of LPC and LPE in our study evidenced that blueberries responded to cold stress. Thus, the changes in blueberry phospholipids during cold storage could be caused by cold stress.

Membrane lipid metabolism also depends on fatty acids (Liu et al., 2011). Linoleic and linolenic acids are unsaturated fatty acids in the cell membrane and are substrates for the specific action of LOX, which is crucial in membrane lipid metabolism. The results of the present study showed that the longer the refrigeration, the lower the content of linoleic and linolenic acids. This might be because long-term refrigeration activated the LOX, which accelerated the rate of decomposition of linoleic and linolenic acids, resulting in the changes observed in cell membrane lipids.

To understand the pitting of blueberries at the cell level, we observed ultrastructural changes in blueberries. Compared with that of the fresh sample, the cell membrane of blueberries with pitting was wrinkled and induced cells plasmolysis, and the structure and function of chloroplasts and mitochondria were destroyed. Therefore, the intracellular macromolecular structure can be severely damaged, eventually leading to tissue collapse and in the appearance of pitting symptoms in blueberries. This result is consistent with previous studies on pepper under cold stress (Sánchez-Bel et al., 2012).

The MRI analysis showed that the water content of blueberries decreased during storage. Dehydration is also a manifestation of CI. Compared with the T_2_ relaxation component in fresh blueberries, the water status in pitted blueberries changed significantly. The relative free water content decreased, and the relative bound water and immobilized water contents increased. This might be due to low temperature stress response, which increases the consumption of free water. In addition, damage to the cytomembrane also exacerbates the loss of free water.

Electrolyte leakage is an important indicator of cell membrane permeability, reflecting cell membrane function and integrity (Chen et al., 2011). Ion leakage is mainly caused by changes in membrane structure and lipid composition (Wongsheree et al., 2009). Therefore, significantly higher electrolyte leakage was observed in the blueberries cold stored for 45 and 60 days throughout shelf life, which is closely related to the changes in membrane structure and lipid composition during long-term refrigeration. Malondialdehyde is a product of membrane lipid peroxidation that is used as an index to reflect the degree of lipid peroxidation (Lin et al., 2014). In the present study, we observed a significant increase in MDA content due to cold storage, especially during shelf life. These data clearly indicated that blueberry membrane lipid peroxidation occurs during refrigeration and it is intensified during shelf life. We also observed that the proline content of blueberries after 45 days of refrigeration was significantly higher than that of other blueberries during the 6-days shelf life (*P* < 0.05). Some studies have pointed out that when plants are exposed to salt, drought, and low-temperature stresses they accumulate highly soluble organic compounds of low molecular weight, such as proline-compatible solutes (Iba, 2002). Obviously, the increase of proline provided strong evidence that blueberries responded to cold stress.

In the present study, the activity of PLD and LOX were significantly increased in the blueberries, which is correlated with the increased permeability of cell membrane and with the degree of membrane lipid peroxidation. Several studies have shown that PLD and LOX are key enzymes in membrane lipid metabolism. They destroy cell membranes mainly by degrading phospholipids and fatty acids in cell membranes, causing disorders in cell metabolism and leading to the decline of cell functions (Paliyath and Thompson, 1987; Paliyath and Droillard, 1992). Some studies showed that PLD and LOX activities increased significantly in pear under low temperature stress (Sheng et al., 2016). In the present study, we also analyzed the expression levels of *PLD* and *LOX* genes. PLDα1 plays an important role in hydrolyzing structural phospholipids into PA under cold stress (Wang, 2002; Rajashekar et al., 2006). However, in the present study, the expressions of *PLDα1* and *PLDα2* were significantly down-regulated in blueberries during cold storage. It is worth noting that *PLDα2* expression increased sharply on day 4 of shelf life after 30 days of cold storage, which might be induced by low temperature. The transcription level of *PLDβ* is higher when plants are exposed to chilling (Zhang et al., 2003; Li et al., 2004). Our results also showed that the expression of *PLDβ* was significantly up-regulated in blueberry during both cold storage and shelf life after 60 days of cold storage. *PLDδ* expression was maintained at a higher level during shelf life after refrigeration, but there were no significant changes during cold storage. Therefore, the activity of PLD seems to be shared by family gene members. Our work consistently showed a significant increase in LOX activity in blueberries refrigerated for 60 days. However, there was a significant decrease in *LOX1* expression in blueberries cold stored for 60 days, which might be due to *LOX1* belonging to the LOX family and to the transcription levels of different gene families differing under abiotic stress.

## Conclusion

The results of the present study showed a correlation between pitting and membrane lipid metabolism in blueberries after refrigeration. First, a significant increase in DGDG, PA, PS, PG, PI, LPC, and LPE and a significant decrease in PC in blueberries after cold storage indicated that the changes in phospholipids during cold storage could be caused by cold stress. Further, we confirmed that dehydration is a manifestation of CI, and the ultrastructure of blueberries demonstrated that membrane integrity and function were destroyed in pitted blueberries. Additionally, the severity of pitting in blueberries was related to relatively higher electrolyte leakage, MDA and proline contents, and the activities of PLD and LOX under low-temperature storage conditions. In conclusion, membrane lipid metabolism plays an important role in blueberry response to cold stress.

## Author Contributions

All authors listed have made a substantial, direct and intellectual contribution to the work, and approved it for publication.

## Conflict of Interest Statement

The authors declare that the research was conducted in the absence of any commercial or financial relationships that could be construed as a potential conflict of interest.
